# Factors contributing to the public proneness towards quacks in Sindh

**DOI:** 10.11604/pamj.2020.37.174.23411

**Published:** 2020-10-21

**Authors:** Rehan Khan, Muhammad Ayaz Mustufa, Saqib Hussain

**Affiliations:** 1 Directorate of Anti-Quackery, Sindh Healthcare Commission, FTC Building, Block C, 2nd floor, Shahrah-e-Faisal, P.O. Box No. 1293, Karachi, Pakistan

**Keywords:** Quack’s impersonation, quack-prone peoples, risk factors

## Abstract

The present study is to explore the factors and reasons behind public proneness towards quacks in the rural areas of Sindh, Pakistan and to manifest the public on how these quacks are duping vulnerable and quackery-prone peoples for financial gain which may induce human lives in life-threatening health conditions. The study also interprets a better understanding of the public needs, especially in the rural areas of the Sindh that may give a hope for deliverance from quacks.

## Commentary

Quacks are usually trained in the rudiments of clinical medicine under licensed doctor tutelage where they hone their skills in primary healthcare services such as compounding and dispensing pharmacy practices and/or they get some informal training as a substitute to phlebotomist. Among their ilk are malpractitioners: (a) individuals who have worked as assistants to qualified physicians, (b) graduated lab technicians who have switched to healthcare, (c) graduated lab technicians working as a substitute to pathologist, sonologist, radiologist and hematologist, (d) inherited midwives without having any formal training or qualifications, (e) Diploma/Bachelor of Homeopathic Medicine and Surgery (DHMS/BHMS) working beyond their scope of practices (i.e. practicing allopathic medicines), and (f) non-qualified person working under the name of a licensed doctor which is called rent-seeking activity. About 6,000 quacks are practicing medicine in Sindh [1]. William H. Gordon in 1967, has categorized the quack-prone peoples into four classes [2]. According to Viola W. Bernard, the biggest reason behind the public vulnerability towards quacks is their inner fears and the quacks give the impression to offer some magical defenses against it [3]. Herein we would like to unveil the peculiar public susceptibility and inclination towards quacks especially in the rural areas and slums of the Sindh, Pakistan. These observations and hypotheses have evolved over time from regular basis anti-quackery campaigns across Sindh by the Directorate of Anti-Quackery-Sindh Healthcare Commission [4]. The following factors may give valuable insights regarding public proneness towards quacks in Sindh, which have not been reported so far:

**Exaggerated claims of quack to cure any disease:** whenever a new discovery is made in the field of medical sciences the quacks venture into it by taking an advantage of inadequate knowledge and lack of interest among public. They pretense to have an effective treatment and vast knowledge of the subject against any disease. At the same time, protecting themselves by emphasizing that there is no guarantee for everyone to be cured. Quacks are creating a persona that entices vulnerable peoples, especially the local youth in Sindh is entrapped at the hands of quacks for their nostrums (herbal supplements) and placebos that will supposedly enhance sexual performance or stamina. According to Unani specialists in Sindh, more than 90 percent of their young clients consult for sexual problems in men. Adult obesity has been rising to an alarming extent [5] and quacks were found involved in practicing obesity medicine too. Usually, less or averaged educated peoples are more prone to quacks because quacks publicize their miraculous healing in a misleading manner among community. “Money back guarantee” is the most popular persuader of the quacks in their advertisements to sell their nostrums as hair tonics to cure baldness. Generally, peoples take this chance to try their luck if they can be cured without spending lots of money on hair transplantation or surgery to promote hair growth (Table 1).

**Table 1:** summary of recommendations at different levels

S.#	Action	Recommendation description
**1**	Public Awareness	Awareness seminars and programs be organized at the district level across the Sindh time to time about the severity and outcomes of quackery practices.
		The local electronic and print media in regional language can play important role in creating awareness among common residents about the harmful aspects of quackery.
		Special programs on the prevalence of potential infectious diseases and health risks by quacks be broadcast from local cable TV network time to time in regional language.
**2**	Sensitizing a wide range of stakeholders	We request the stakeholder be more communicative with us in the field and with DAQ-SHCC's inspection & enforcement teams that will help us to ease our collaboration with law and enforcement agencies and local authorities in order to get deliverance from the menace of quacks.
**3**	Governing bodies at provincial and national levels	Healthcare regulatory authorities at the provincial and national level should support the cause and monitor quality of care and health care professional's databases to ensure influential and political bias of licensure.
**4**	Social mobilization tools	Mobilization of the community by identifying key persons such as retired health professionals, local NGO's, social activists, and district health officers who can act as a contact person between locals and the administration.

**Note:** the opinions expressed in these recommendations are the responsibility of the authors and do not necessarily reflect the official policy of the Sindh Healthcare Commission (SHCC).

**Sensational claims of quack to surprise their clients:** to retain the faith in their clients, quacks tend to make them surprise by their fake inspirational success stories. Oftentimes they are observed to claim that they can diagnose many diseases just by feeling the pulse. In order to do so, they hold patient´s wrist briefly and conclude their determinations. They are usually aware of the recent scientific advices about some diseases and selling their clients a false hope at a high price. They use to tell their clients that thousands of peoples have been cured at the hands of their forefathers and they have some inherited magical cures, discoveries and some unveiled secrets that is unknown to others. Quacks know, they can't solve various health problems and issues, but it's quacks nature to clutch at straws. It is often observed that senior citizens due to their aching muscles are more vulnerable to quacks and are seeking some magical cures to soothe their aching muscles.

**Lack and unavailability of a licensed doctor when healthcare services are needed:** the governmental organizations in Sindh, such as, Basic Health Unit (BHU), People's Primary Healthcare Initiative (PPHI), Rural Health Centers (RHC), District Headquarters (DHQ) and Taluka (THQ) hospitals provide specialist care in the morning hours only and are situated at a distance of about 30-35 km from many populated rural areas. Hence, lack and unavailability of substitute qualified doctor in late evening/night emergency situation are another factor that influences quackery-prone peoples. It is observed that desperation and vulnerability set in when someone is suffering from the highly debilitating injury or illness and a licensed doctor is not accessible at that time. Under such situations, everything can be believed that sounds hopeful. This desperation causes the local to begin trusting the quacks. Therefore, locals of rural areas depend on quacks for accessible and affordable healthcare. Nowadays, quacks are available on motorbikes (mobile quacks) to head out after patients in the rural areas and are immediately available in emergency situations. These mobile quacks cycle around villages, so that they are easily accessible to the locals.

**Building strong relationship with patients without money:** the entrepreneurial mindset and behavior of the quacks contribute a significant role in building a quack-patient relationship. The feudalism has crept so much in the rural areas of Pakistan, and they are the most influential in their respective areas. So, quacks are usually giving their families a cost-free healthcare services, consequently the locals are compulsive to go quacks. Quacks usually participate in social gatherings a lot and they almost know every house in many villages. Sometimes, they treat their patients without money or on account in their impoverished community, which may have been to encourage their clients not to switch to a competitor. Quacks are informally trained under tutelage of a qualified doctor, and the doctors sometimes send them to their client´s home for dressing change or insulin injection etc. Sometimes, these informally trained staff are also assisting the doctors to lighten patient´s burden. This is how patient´s trust and relationship are developed in their dispensers, compounders and lab technicians. So, they take an advantage of the public trust to continue their malpractices for the rest of his/her life as a successor of the well-regarded and renowned doctor. Another factor that induces quackery-prone peoples is their trust in quacks because they are being treated by them for the last several years.

**Symptomatic treatment to immediately relief the patients:** this has been a general psyche of the locals of rural areas and slums that they only believe in quick-fix or immediate effect of the treatment. If they are not administered injections and/or given intravenous fluids, they are not satisfied at all by the doctor. On the other hands, quacks almost always administer injections to their patients, whether for headache or fever, and give intravenous fluids to line their pockets without realizing the basic cause of the disease. Quack´s palliative treatment/measures typically include mix medicines such as broad-spectrum antibiotic, injections and an anti-inflammatory to cover all common diseases found among community of their respective areas.

**Recommendation:** it is a collective duty and responsibility of the accountable institutions, medical societies, most importantly the community, health professionals, medical licensing boards, stakeholders, law and enforcement agencies, and regulatory bodies in the country need to take some urgent and practical steps on its numerous failures and begin to systematically proffer solutions alongside an efficient implementation machinery.

**Conclusion:** the growing predilection for quacks among rural residents has been a serious threat to public health. The Government shall improve healthcare infrastructure in the rural areas of Sindh so that doctors will be encouraged to go and practice over there. This is how the primary and secondary healthcare level will be strengthened with optimal health care facilities and adequate staff. However, public education, awareness, and sensitization is recommended to effectively combat the menace of quackery for the sake of public health and to save the status of the highly regarded medical profession. It is illegal to sell intoxicating drugs, antibiotics, painkillers and injections to the quacks in most parts of Pakistan, but the law is rarely enforced.

## References

[1] The Express Tribune. Over 5,000 quacks traced across Sindh.

[2] Gordon WH (1967). Why people go to quacks. Phys Ther.

[3] Viola WB (1965). Why peoples become the victims of medical quackery. Am J Public Health Nations Health.

[4] Sindh Healthcare Commission. Scope & legal mandate.

[5] The Organization for Economic Cooperation and Development (OECD) Obesity update-2017.

